# 肺癌与TTF-1分子标志物研究进展

**DOI:** 10.3779/j.issn.1009-3419.2014.06.10

**Published:** 2014-06-20

**Authors:** 晓晓 葛, 丽岩 姜

**Affiliations:** 200030 上海，上海交通大学附属胸科医院呼吸科 Department of Pulmonary, Shanghai Chest Hospital, Shanghai Jiaotong University, Shanghai 200030, China

**Keywords:** 肺肿瘤, 甲状腺转录因子-1, 生物标志物, Lung neoplasms, Thyroid nuclear factor-1, Biological markers

## Abstract

肺癌是全球癌症相关死亡的主要病因之一。多数的肺癌患者确诊时已处于晚期，中位生存期为1年左右，5年生存率不足16%，预后极差。近年来的研究热点主要集中于涉及致癌过程中新的生物因子的潜在作用。最近研究表明，甲状腺转录因子（thyroid transcription factor-1, TTF-1）是肺癌的一种特异性的谱系生存癌基因。在肿瘤进展过程中，TTF-1的生物学活性和临床功能表现出相反的作用。在此，本文总结了TTF-1在肺癌的发生、发展、诊断及预后方面的作用，以期为TTF-1作为肺癌新型生物标记物的作用提供见解。

## Abstract (en)

Lung cancer is the leading cause of cancer-related death worldwide. The majority of cases, the diagnosis is made at an advanced stage. The prognosis is poor with a 5-year survival rate less than 16% and the median overall survival 1 year or so. In recent years, research has focused on the potential role of new biological factors involved in the carcinogenic process. It's well known that thyroid transcription factor 1 (TTF-1) was a special lineage-survival oncogene in lung cancer according to recent researches. It has become apparent that TTF-1 expression has biological and clinical functions in the opposite direction that act in tumor progression. Herein, we summarize the role of this transcription factor in the development, diagnosis, and prognosis of lung cancer in the hope of providing insights into the utility of TTF-1 as a novel biomarker of lung cancer.

近半个世纪以来，世界各国的肺癌发病率和死亡率均有明显增高的趋势。目前，肺癌的发病率和死亡率在世界范围内居癌症之首^[1-3]^。据我国卫生部和科技部第三次居民死亡原因抽样调查结果显示，过去的30年内，肺癌的发病率上升了46.5%，上升幅度居各类型肿瘤之最。目前肺癌死亡人数占全部恶性肿瘤死亡人数的22.7%，已替代肝癌成为我国恶性肿瘤死亡原因之首。中国男性的肺癌发病率为63.51/10万人，女性的肺癌发病率为46.4/10万人，均分别位于恶性肿瘤发病率的首位。据世界卫生组织预测，到2025年，我国每年新增的肺癌病死例数将超过100万，患病人数将位于世界首位^[4]^。非小细胞肺癌（non-small cell lung cancer, NSCLC）是肺癌的主要组织类型，约占已确诊的原发性肺癌总体的85%^[5]^。多数NSCLC确诊时已处于晚期，中位生存期为1年左右，5年生存率不足16%，预后极差。即使早期可以手术的患者，仍有多数患者在5年内出现肿瘤复发或转移^[6]^。在这种情况下，肺癌的诊断、治疗及预后就不能单纯依靠现行的肺癌组织病理分型和TNM（tumor-node-metastasis）分期。因此，分子病理学领域也逐渐成为肺癌的研究热点。其中，部分研究致力于分析新的生物因子在致癌过程中的潜在作用。随着研究的不断深入，越来越多的数据证明，甲状腺转录因子（thyroid transcription factor-1, TTF-1）在肺癌发生发展中起到致癌作用，TTF-1调控肺癌的发生、发展及预后，与疾病的诊断、治疗、预后亦有一定的关联。本文就TTF-1作为肺部肿瘤的谱系特异性生存基因的相关研究进展进行综述。

## 1 TTF-1结构和功能

TTF-1的研究始于1989年，Civitareale等^[7]^通过动物实验发现TTF-1可以调节甲状腺球蛋白基因的特异性表达，为甲状腺球蛋白的特异性转录因子，从而命名为甲状腺转录因子-1。随后接踵而至的大量研究主要集中于阐释TTF-1在甲状腺和肺发生发育中的作用。

TTF-1也称为NKX同源框-1（NK2 homeobox 1, NKX2-1），是NKX2转录基因家族的成员之一。TTF-1位于人类染色体14q13.3，由三个外显子和两个内含子组成的单基因编码的含有一个同源结构域的转录因子。人类、小鼠、大鼠及其他物种的TTF-1所含氨基酸序列约98%是相同的，提示其序列高度保守。针对*TTF-1*基因的DNA片段分析显示，在TTF-1的第1个外显子和第1个内含子中存在2个独立区，这与调节肺组织上皮细胞基因的启动活性有关^[8]^。人类TTF-1的三维结构含DNA结合域及转录活性域。DNA结合域为HD结构，由60个氨基酸残基组成3个螺旋，螺旋Ⅰ、Ⅱ与DNA的碳骨架结合，螺旋Ⅲ在进化上高度保守，能识别特定的DNA序列, 该序列的核心是5’-2CAAG2-3’。TTF-1启动子的活性是由肝细胞核因子-3（hepatocyte nuclear factor 3, HNF-3）又称为FOXA（forkhead box A），特异性蛋白1（specificity p rotein 1, Sp1），特异性蛋白3（specificity p protein, Sp3），GATA转录因子26（GATA transcription factor 26）及HOXB3（homeobox B3）转录因子共同调节完成^[9]^。

TTF-1编码的蛋白含有371个氨基酸，是相对分子量为38 kDa的核蛋白，生理情况分布于甲状腺、呼吸道上皮的Ⅱ型Clara细胞及部分大脑前腹侧核等，调控甲状腺、肺和中枢神经系统中基因的选择性表达^[10]^。人类单纯性缺乏TTF-1可导致甲状腺功能低下、呼吸功能不良，易引起肺部的反复感染，这提示最佳表达水平的TTF-1表达对维持甲状腺和肺正常功能的重要性。此外，研究^[11]^表明TTF-1参与调节多种基因表达，在肺上皮组织的形态发生发育及分化过程中起到重大作用。

## 2 TTF-1在肺组织的表达及功能

### 2.1 正常肺组织TTF-1的表达及功能

TTF-1生理状态下主要分布于胎肺、甲状腺及部分大脑前腹侧核。出生后，TTF-1在一些特殊的上皮细胞中表达，如肺泡的Ⅱ型细胞、Clara细胞和支气管基地细胞，然而肺Ⅰ型上皮细胞无TTF-1的表达。在胚胎发育过程中，TTF-1是受严格时空调控进行表达，最早可检测到TTF-1表达是在大脑的前腹侧内胚层细胞，此后，TTF-1在由肺原始细胞产生的气管的祖细胞上广泛表达，最终TTF-1表达多限制于肺外周的终末呼吸单元（terminal respiratory unit, TRU）^[12, 13]^。在正常的肺上皮细胞发育过程中，TTF-1首先是在胞浆中合成，然后被转运至细胞核中。TTF-1在TRU的表达与其调节肺泡的发育和肺表面活性蛋白的作用一致^[14, 15]^，TTF-1调控肺泡表面活性蛋白A（surfactant protein A, SP-A）、肺泡表面活性蛋白B（surfactant protein B, SP-B）、肺泡表面活性蛋白C（surfactant protein C, SP-C）及Clara细胞分泌蛋白（Clara cell secretion protein, CCSP）的表达^[16]^。研究发现，利用基因敲除手段使得小鼠上的*TTF-1*基因表达沉默，从而发现小鼠的肺、甲状腺等组织器官发育畸形，同时伴随调节甲状腺和肺的特异性基因作用失调。另一项研究结果显示，夹闭小鼠的支气管，在肺泡塌陷或感染的区域内，TTF-1几乎不表达，然而去除这些不良的因素，这些肺泡又重新呈现TTF-1的表达，这提示TTF-1与肺泡上皮细胞的形成及肺泡表面蛋白的形成有潜在的调节作用。

### 2.2 肺癌组织中TTF-1的表达及功能

研究^[12, 17]^证明，TTF-1与多种转录因子互相作用从而以特异性的方式调节细胞或组织的多种分化和发育功能。同时研究发现，在肺癌中，TTF-1在促进肿瘤细胞分化和形态发育中起到关键作用。

首先，在TTF-1蛋白的表达谱中，研究发现在不同类型的肺癌组织中，TTF-1的表达也各有不同。例如，在肺腺癌和小细胞型肺癌中，TTF-1高表达；然而在大细胞性肺癌和肺鳞癌中，TTF-1高表达较为少见。2003年Tan等^[18]^报道了126例Ⅰ期-Ⅲ期NSCLC患者组织中不同病理亚型的TTF-1表达状况，结果显示，68%（51/75）的腺癌呈现TTF-1高表达，然而仅仅只有21%（9/43）的鳞癌患者存在TTF-1表达。Yatabe等^[12]^研究显示，TTF-1阳性表达多于女性、非吸烟患者。Pelosi等^[19]^发现，TTF-1的阳性表达与肺腺癌的直径相关，在直径小于3 cm的腺癌组织中，其表达阳性率较高（*P*=0.08）。此外，还有研究^[20]^提示，存在*p53*和*KRAS*突变的肺癌组织中罕见TTF-1的表达；粘液性腺癌组织存在*KRAS*突变，则无*EGFR*突变及TTF-1蛋白阳性表达。Kaufmann等^[21]^从138例原发性肺癌患者的组织亚型中发现，75%的非粘液性肺癌组织存在TTF-1表达，与之呈鲜明对比的是，仅有10%的粘液性腺癌样本中有TTF-1表达。Winslow等^[22]^2011年在《Nature》杂志上发表的文章显示，低分化的肺癌中TTF-1的表达多为阴性；在原发性和转移性肿瘤来源的肺癌细胞内进行TTF-1的功能扩大和失效实验发现，TTF-1可以控制肿瘤的分化程度及限制恶性细胞转移的潜能；在观察TTF-1下调至肿瘤分化程度降低时发现此时的肿瘤细胞的播种能力增强，恶性转移倾向增加。因此，即使在同一类型的肿瘤中，对细胞的致癌性及抑癌性功能证明TTF-1起到双重调节作用。

其次，在*TTF-1*基因研究领域中发现，肺腺癌中存在TTF-1的表达及基因的拷贝^[17, 23, 24]^，研究提示，干扰TTF-1的表达，可导致肺腺癌细胞生长受抑和凋亡。Weir^[25]^的研究发现，肺腺癌中常见的遗传学改变之一是12%的肺腺癌中存在*TTF-1*基因的高水平扩增。Bai等^[26]^在92例肺癌及癌旁组织中，通过PCR比对发现16%肺癌组织中存在*TTF-1*基因的错义和同义突变，提示检测*TTF-1*基因突变在进一步探索肺癌的发病机制中具有重要意义。现有研究^[27]^发现*TTF-1*是肺癌的谱系特异性致癌基因，调节肺癌的发生发展，与肺癌细胞的生长和存活密切相关。

适量的TTF-1表达在维持肺等重要器官的发育上起到调节作用，过量的TTF-1的扩增及表达则参与肺癌的发生、发展，与肺癌的诊断、治疗及预后存在一定的关系。

## 3 肺癌中TTF-1的诊断价值

TTF-1是近年来发现的一个新的肿瘤分子标记物，是肺腺癌高敏感性及特异性的分子标记物，目前已证实其在肺癌的诊断和鉴别诊断方面具有非常明显的应用价值。Stenhouse等^[28]^的研究中确立了TTF-1的阳性表达主要在于外周型的肺泡等终末端气道，研究发现，106例非肺腺癌的腺癌组织中TTF-1基本不表达；73%的肺腺癌中出现TTF-1的阳性表达；粘液性肺癌或分化程度较低的肺癌中TTF-1的表达较低。Wu等^[29]^研究提示，在80例原发性肺癌组织中，89.7%肺腺癌存在TTF-1表达，具有较高的敏感性和特异性，在临床病理学诊断，尤其是小标本病理诊断时，TTF-1可作为肺腺癌的一个比较敏感和特异的免疫组化标记。一项针对转移性腺癌及原发性肺腺癌特异性标记物的研究^[30]^发现，腺癌细胞中TTF-1的阳性表达率极高，联合Napsin A和TTF-1免疫组化指标后发现，120例肺腺癌中有95例（79.2%）呈现Napsin A及TTF-1双阳性表达。这些研究提示TTF-1的阳性表达是肺腺癌特异的免疫组化诊断标志物，有助于转移性腺癌和原发性肺腺癌的鉴别。

## 4 肺癌中TTF-1的治疗作用

2009年，Hsu等^[31]^研究发现，*TTF-1*和*NKX2-8*基因共活化的肺癌细胞株显示对以顺铂为主NSCLC标准治疗显示耐药。迄今为止，罕有研究发现TTF-1和患者对EGFR-TKI药物之间的敏感性。Wislez^[32]^2010年的研究证明在细支气管肺泡癌中发现，95%的非粘液肺癌中存在TTF-1高表达，然而27%的粘液性肺癌中无表达；此外，他的研究显示非粘液肺癌对EGFR-TKI药物表现的更敏感。Somaiah等^[33]^报道了TTF-1与肺癌的相关研究结果，TTF-1表达阴性的患者罕见*EGFR*突变，TTF-1的特异性及灵敏度分别为36.4%和99.1%。这也意味着肺癌族中TTF-1高表达可能是预测肺癌*EGFR*基因突变的一个良好的免疫组化指标，可以推测TTF-1高表达则*EGFR*突变率高，可能是肺癌患者服用EGFR-TKI类药物的优势人群特征之一。然而TTF-1高表达是否表示对EGFR-TKI类药物更敏感有待后续机制研究证明。基于这些数据，有理由相信根据TTF-1表达、NSCLC的组织亚型和*EGFR*突变状态等分子病理学及组织病理学特征，从而制定出合理的治疗策略。

## 5 肺癌中TTF-1的预后价值

尽管近几年中，肺癌患者的5年生存率有3%-5%的提高，然而仍有许多患者并未获益。因此，对于现有体系的治疗，首先去特别评估NSCLC患者生存是第一位。这需要聚焦于临床研究，期待可提供个体化治疗方案。此外，利用现有科学技术，研究肺癌的分子发病机制也相当重要，在疾病进展时，TTF-1有可能是分子治疗干预的靶向标记物。目前关于TTF-1是否影响肺癌的预后的研究已经开展，但是在TTF-1阳性表达与生存的关系存在很大的分歧。

Berghmans等^[34]^认为，TTF-1阳性表达与患者预后较高之间的关系有统计学意义，尤其是在早期局限期肺腺癌中。Nakamura等^[35]^认为由于TTF-1生理状况下参与调节肺上皮组织发育、分化，因此可以推测肿瘤中仍然保留的TTF-1表型可能代表着肿瘤尚保留一定的正常分化能力，相对来说其侵袭力较弱。Myong^[36]^研究发现，TTF-1表达与细胞增殖抗原（Ki67）表达呈负性相关，意味着TTF-1表达水平越高，瘤细胞增殖率越低，提示患者的预后越好。这些研究提示，TTF-1阳性表达可能代表肺癌患者的生存时间较长。

同时，也有学者认为，TTF-1阳性表达是肺癌患者生存期的不良预后因素，如Lee等^[37]^分析118例术后原发性肺腺癌预后，得出*EGFR*或*TTF-1*基因扩增是患者无疾病生存期的独立不良预后因素。Pelosi^[19]^研究结果显示，在肺腺癌中，TTF-1有可能通过增加肿瘤新生血管从而加速肺癌的进展。Yoon等^[38]^利用实时PCR的方法检测79例肺癌术后患者血液里循环肿瘤细胞（circulating tumor cells, CTC）的TTF-1及CK19的表达，结果显示，在NSCLC患者的CTC中，TTF-1的转录明显高于CK19；TTF-1的阳性表达较阴性表达患者的疾病无进展生存时间更短（*P*=0.004）。

以上研究提示TTF-1与预后之间的结果相对不统一。这可能是与TTF-1在肺癌中的调控机制尚不明确，或可能与在肺癌的不同阶段TTF-1的作用不同相关。总之，今后应加强以TTF-1为中心的调控网络的分子学研究，从而可以更为准确的探讨TTF-1与肺癌预后之间的关系。

## 6 结论

综上所述，以往的研究重点侧重于TTF-1在肺生理学领域的作用。然而随着科学技术的进一步提高，新的证据更关注与TTF-1在肺癌发生发展中的调节作用。据以往文献，TTF-1在肺癌的发病机制中似乎起着双刃剑的作用，但是具体作用机制不甚明了。因此未来的研究应该更加严谨的探测*TTF-1*基因的更多的下游分子，从而全方面的了解以TTF-1为中心的肿瘤调控网络。此外，研究TTF-1对不同组织亚型的肺癌，尤其是肺腺癌的预后影响因素，以及这些因素对患者化疗后的疗效评价及生存将有助于开发新的治疗策略。
